# Long Non Coding RNA MALAT1 Promotes Tumor Growth and Metastasis by inducing Epithelial-Mesenchymal Transition in Oral Squamous Cell Carcinoma

**DOI:** 10.1038/srep15972

**Published:** 2015-11-02

**Authors:** Xuan Zhou, Su Liu, Guoshuai Cai, Lingping Kong, Tingting Zhang, Yu Ren, Yansheng Wu, Mei Mei, Lun Zhang, Xudong Wang

**Affiliations:** 1 The Department of Otorhinolaryngology and Maxillofacial Oncology, Tianjin Medical University Cancer Institute & Hospital, National Clinical Research Center for Cancer, Key Laboratory of Cancer Prevention and Therapy, Tianjin, 300060, China.; 2 Department of Genetics, Geisel School of Medicine at Dartmouth, Hanover, NH, 03755, USA.; 3 Department of Oral and Maxillofacial Surgery, Stomatological Hospital, Tianjin Medical University, Tianjin 300070, China; 4 Tianjin Research Center of Basic Medical Science, Tianjin Medical University, Tianjin 300070, China

## Abstract

The prognosis of advanced oral squamous cell carcinoma (OSCC) patients remains dismal, and a better understanding of the underlying mechanisms is critical for identifying effective targets with therapeutic potential to improve the survival of patients with OSCC. This study aims to clarify the clinical and biological significance of metastasis-associated long non-coding RNA, metastasis-associated lung adenocarcinoma transcript 1 (MALAT1) in OSCC. We found that MALAT1 is overexpressed in OSCC tissues compared to normal oral mucosa by real-time PCR. MALAT1 served as a new prognostic factor in OSCC patients. When knockdown by small interfering RNA (siRNA) in OSCC cell lines TSCCA and Tca8113, MALAT1 was shown to be required for maintaining epithelial-mesenchymal transition (EMT) mediated cell migration and invasion. Western blot and immunofluorescence staining showed that MALAT1 knockdown significantly suppressed N-cadherin and Vimentin expression but induced E-cadherin expression *in vitro*. Meanwhile, both nucleus and cytoplasm levels of β-catenin and NF-κB were attenuated, while elevated MALAT1 level triggered the expression of β-catenin and NF-κB. More importantly, targeting MALAT1 inhibited TSCCA cell-induced xenograft tumor growth *in vivo*. Therefore, these findings provide mechanistic insight into the role of MALAT1 in regulating OSCC metastasis, suggesting that MALAT1 is an important prognostic factor and therapeutic target for OSCC.

Head and neck squamous cell carcinoma (HNSCC) is one of the most prevalent tumors in the world with over 500,000 cases diagnosed annually. Oral squamous cell carcinoma (OSCC), a subset of HNSCC, is characterized by a high risk of lymphnode metastasis and local invasiveness^1^. Despite of radical surgery combined with radiation, chemotherapy and targeted therapy, OSCC has a 5-year survival rate of about 50%^2^. Thus, it’s urgent to find notable prognostic factors or metastatic predictors and therapeutic targets in OSCC.

Epithelial-mesenchymal transition (EMT) endows metastatic properties upon cancer cells to promote migration, invasion, and subsequent dissemination and it is mainly characterized by loss of E-cadherin expression^3^,^4^. Up to now, it is unclear which subpopulation of OSCC patients will develop lymphnode or distant metastasis. Recent researches indicate that multiple factors, including NF-κB^5^, conical Wnt/β-catenin^6^, PI3K/Akt^7^, and EGFR signaling pathways^8^ may regulate the EMT process and mediate metastasis cascade in cancer cells.

Long noncoding RNAs (lncRNAs) are a class of non-protein coding transcripts longer than 200 nucleotides. Although increasing evidence describes lncRNAs as notable molecular markers involved in regulating gene expression and cancer progression, our understanding of its role in cancer metastasis is very limited^9^,^10^. Recently several lncRNAs are validated to be associated with cancer metastasis and regulated by oncogenic or tumor suppressor pathways^10^. Metastasis-associated lung adenocarcinoma transcript 1 (MALAT1), a highly conserved mammals’ lncRNA, is the transcript of noncoding nuclear-enriched abundant transcript 2 (NEAT2),and is specifically retained in nuclear speckles to modify pre-mRNA processing^11^. MALAT-1 was first identified as prognostic parameters for patient survival in stage I non-small cell lung cancer (NSCLC)^12^. Recent researches suggest that MALAT1 level is positively correlated with clinical stages and serves as an oncogene in esophageal squamous cell carcinoma^13^, glioma^14^, renal cell carcinoma^15^and etc. Mechanistic investigations showed that MALAT1 was transcriptionally activated by c-Fos or Sp1 in renal cell carcinoma and lung cancer cells^15^,^16^. Another post-transcriptional regulation mechanism of MALAT1 by miR-101 and miR-217 exists in ESCC cells and this post-transcriptional silencing of MALAT1 could significantly suppress the proliferation of ESCC cells through the arrest of G2/M cell cycle^17^. MALAT1 has been shown to regulate some metastasis-associated genes in colon cancer, non-small cell lung cancer (NSCLC), glioma, and myeloma^11^,^18^,^19^. However, the role of MALAT1 in mediating the metastasis of OSCC has not been well investigated.

In the current study, by using gain- or lose-of-function assays, we validated that MALAT1 contributed to EMT-mediated metastasis in OSCC by modulating the activation of β-catenin and NF-κB pathways.

## Results

### MALAT1 overexpression was associated with poor prognosis in OSCC

**Figure 1:**
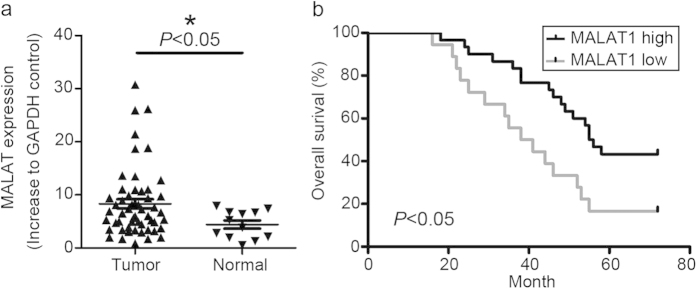
Expression of MALAT1 in human oral squamous cell carcinoma. (**a**) MALAT1 was overexpressed in 54 OSCC samples (*P* < 0.05). (**b**) Kaplan–Meier survival curve analysis indicated that OSCC patients with lower MALAT1 expression had prolonged survival time compared with patients with high levels of MALAT1 (log-rank test, *P* < 0.05).

Post-surgery pathological diagnosis indicated that, 10 of the 54 OSCC cases were well-differentiated, 20 cases were moderately differentiated and 24 cases were poorly differentiated tumors. First, we defined the MALAT1 expression status in all the 54 OSCC samples and 10 non-tumor normal oral mucosa samples. QPCR data indicated that MALAT1 expression in tumor (8.318 ± 0.88) is higher than in the normal mucosa samples (4.413 ± 0.757) (*P* < 0.05, Fig. 1a). No statistical difference in MALAT1 expression was detected by tumor differentiation status (data not shown). Second, according to the median MALAT1 expression, we divided the 54 OSCC cases into high and low MALAT1 expression groups. Kaplan-Meier survival analysis showed that patients with low MALAT1 expression (n = 35) had significantly increased overall survival compared with patients with high MALAT1 expression (n = 19, *P* < 0.05; Fig. 1b).

### Inhibition of MALAT1 attenuated OSCC invasion and migration *in vitro*

**Figure 2:**
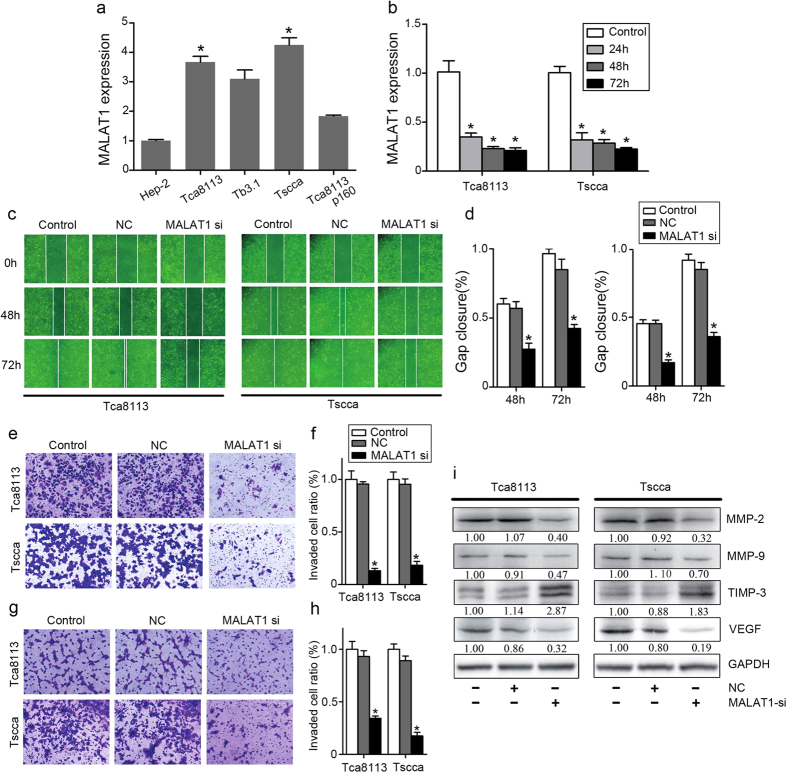
Inhibition of MALAT1 attenuated OSCC invasion and migration *in vitro*. (**a**) Tscca and Tca8113 cells displayed highest expression levels of MALAT1 among the HNSCC cell lines. (**b**) MALAT1 siRNA transfection inhibited effectively MALAT1 expression in Tscca and Tca8113 cells (*P* < 0.05). (**c**,**d**) MALAT1 siRNA significantly inhibited Tscca and Tca8113 cell migration determined by wound healing assay (*P* < 0.05). (**e**,**f**) MALAT1 siRNA significantly inhibited Tscca and Tca8113 cell migration and invasion determined by transwell assays with matrigel (*P* < 0.05). (**g**,**h**) MALAT1 siRNA significantly inhibited Tscca and Tca8113 cell migration determined by transwell assays without matrigel (*P* < 0.05). (**i**) MALAT1 siRNA significantly inhibited the expression of MMP2/9, and VEGF, but increased the expression of TIMP-3 in Tscca and Tca8113 cells determined by western blots analysis (*P* < 0.05). The gels were crapped, but those gels were run under the same experimental condition.

We measured the MALAT1 expression in Tscca, Tca8113P160, Tca8113, Hep-2 and Tb3.1 cells by qPCR. Tca8113 and Tscca OSCC cells showed higher expression levels of MALAT1 than the other 3 cell lines(*P* < 0.05, Fig. 2a).Thus, we chose Tca8113 and Tscca for further studies. In order to determine inhibition of MALAT1 might suppress OSCC cell invasion and migration *in vitro*, we transfected Tca8113 and Tscca OSCC cells with MALAT1 siRNA and a negative control oligonucleotide. At 24h, 48h and 72h after transfection, there was a significantly decrease of MALAT1 expression in both cell lines (*P* < 0.05, Fig. 2b). As indicated by the wound-healing assay, MALAT1 knockdown inhibited Tca8113 and Tscca cell migration ability compared with negative control or scramble transfected cells (*P* < 0.05, Fig. 2c,d). The transwell assay with matrigel (*P* < 0.05, Fig. 2e,f) or without matrigel (*P* < 0.05, Fig. 2g,h) revealed that in MALAT1 knockdown cells, invaded Tca8113 and Tscca cell numbers decreased significantly.

Overexpression of Matrix metalloproteinases (MMP-2/9) were believed to represent a strong invasion capability by degrading extracellular matrix (ECM) in cancer cells. Tissue inhibitors of tissue inhibitors of metalloproteinases (TIMPs) control MMP activities to minimize matrix degradation^20^,^21^. To further validate the role of MALAT1 in modulating OSCC invasion, we measured the expression levels of MMP-2, MMP-9 and TIMP-3. As shown in Fig. 2i, Tca8113 and Tscca OSCC cells displayed suppressed MMP-2/9 and VEGF expression but upregulated TIMP-3 expression after MALAT1 knockdown(*P* < 0.05). These results confirmed that MALAT1 inhibition downregulated MMP2/9 expression, and suppressed OSCC cell invasion and migration *in vitro*.

### Inhibition of MALAT1 suppressed OSCC EMT *in vitro*

**Figure 3:**
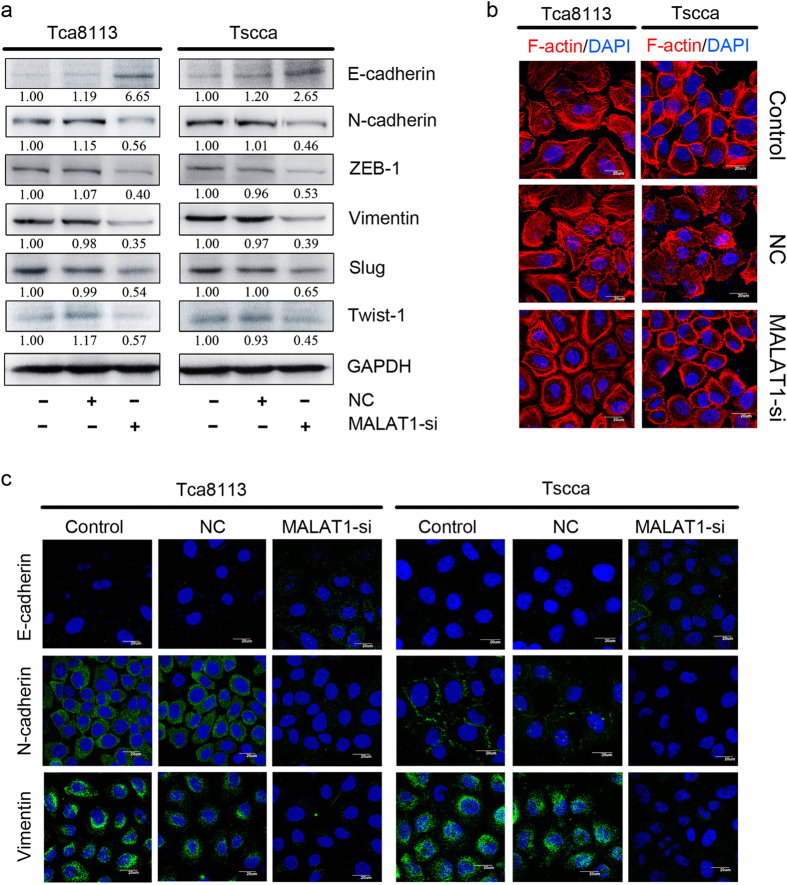
Inhibition of MALAT1 suppressed EMT in OSCC *in vitro*. (**a**) MALAT1 siRNA significantly increased the expression of E-cadherin and inhibited the expression of N-cadherin, ZEB1, Vimentin, Slug, and Twist-1 in Tscca and Tca8113 cells determined by western blots analysis(*P* < 0.05). The gels were crapped, but those gels were run under the same experimental condition. (**b**) F-actin staining showed a stress-fiber pattern in control or NC siRNA treated cells, whereas a cortical pattern in MALAT1 siRNA-treated cells by immunofluorescence staining. (**c**) MALAT1 siRNA significantly upregulated E-cadherin and downregulated N-cadherin and Vimentin in Tscca and Tca8113 cells determined by immunofluorescence staining (original magnification: 1000×).

Previous studies have established that EMT is required for metastasis in multiple human epithelium cancers. Consequently, we measured EMT markers by western blot to evaluate whether MALAT1 could promote EMT-mediated OSCC invasion. Specifically, loss-of-function of E-cadherin was believed to initiate EMT and human cancer metastasis^22^. In Tca8113 and Tscca cells, MALAT1 silencing induced E-cadherin by 2.65–6.65 folds (*P* < 0.05), and suppressed N-cadherin by 1.79–2.17 folds (*P* < 0.05) comparing to negative control and scramble cells (Fig. 3a). Similarly MALAT1 silencing suppressed Vimentin expression by 0.4–0.53 in both the two cell lines (*P* < 0.05, Fig. 3a). Moreover, notable transcription factors like Slug, Zeb-1 and Twist-1 expression were significantly inhibited in MALAT1 siRNA treated Tca8113 and Tscca cells (*P* < 0.05, Fig. 3a).

To further explore the effect of targeting MALAT1 on EMT, IF was employed to analyze the expression of epithelial and mesenchymal markers, as shown in Fig. 3b,c. Treatment of MALAT1 siRNA resulted in increased expression of E-cadherin, but decreased expression of Vimentin and N-cadherin, compared to that in control cells. We next examined the morphological changes of F-actin, which was required for invadopodia formation^23^. In negative control and scramble OSCC cells, F-actin presented a strong cortical pattern, suggesting mesenchymal features and high movement capability (Fig. 3b). While in MALAT1 siRNA treated cells, F-actin presented relative stress fiber pattern, suggesting significantly inhibitory effect on mesenchymal features and cell movement capability. Taken together, these data indicated that MALAT1 silencing could dramatically affect the EMT and rearrangement of cytoskeletal proteins in OSCC cells *in vitro*.

### Inhibition of MALAT1 reduced the activation of β-catenin and NF-κB signaling pathways in OSCC

**Figure 4:**
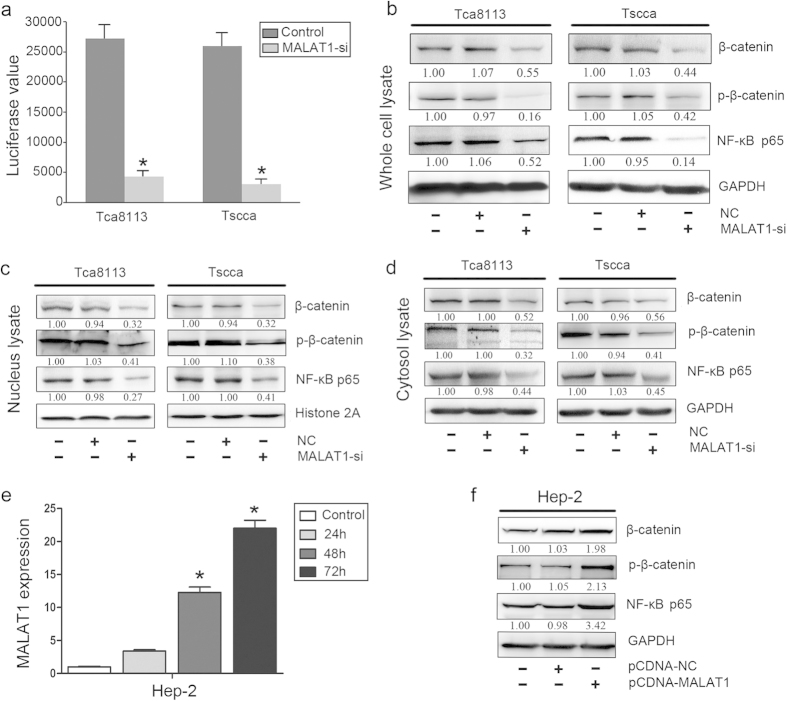
Inhibition of MALAT1 reduced the activity and level of β-catenin and NF-κB in OSCC. (**a**) A TOP flash reporter assay indicated that MALAT1 siRNA treatment inhibited β-catenin transcriptional activity in Tscca and Tca8113 cells (*P* < 0.05). (**b**) MALAT1 siRNA treatment inhibited β-catenin, p-β-catenin and NF-kBp65 expression in whole cell lysate of Tscca and Tca8113 cells (*P* < 0.05). (**c**) MALAT1 siRNA treatment inhibited β-catenin, p-β-catenin and NF-kBp65 expression in nucleus lysate of Tscca and Tca8113 cells (*P* < 0.05). (**d**) MALAT1 siRNA treatment inhibited β-catenin, p-β-catenin and NF-kBp65 expression in cytosol lysate of Tscca and Tca8113 cells (*P* < 0.05). (**e**) PCDNA3-MALAT1 vector transfection increased MALAT1 expression in Hep-2 cells with a relative low level of MALAT1 expression (*P* < 0.05). (**f**) PCDNA3-MALAT1 vector transfection increased the expression of β-catenin, p-β-catenin and NF-kBp65 in whole lysate of Hep-2 cells (*P* < 0.05). The gels were crapped, but those gels were run under the same experimental condition.

Since β-catenin and NF-κB pathways were reported to be the key regulators in EMT, we consequently explored whether MALAT1 gain- or loss- of function could mediate EMT in OSCC through β-catenin and NF-κB pathway. We measured whether MALAT1 knockdown could affect β-catenin transcriptional activity by the TOP flash reporter assay. Compared with negative control and scramble cells, remarkably decreased activity of the β-catenin/TCF4 was observed in both MALAT1 siRNA treated Tca8113 and Tscca cells (*P* < 0.05, Fig. 4a). Whole cell lysate analysis indicated that β-catenin and phosphorylated β-catenin level were decreased in both cell lines (*P* < 0.05, Fig. 4b). Additionally, NF-κBp65, the activation form of NF-κB, displayed a decreased expression level in MALAT1 siRNA treated OSCC cells (*P* < 0.05, Fig. 4b). Cell components of protein analysis indicated that β-catenin, p-β-catenin and NF-κBp65 were suppressed significantly in nucleus and cytoplasm (*P* < 0.05, Fig. 4c,d). To further validate the effect of MALAT1 on β-catenin and NF-κB, we introduced MALAT1 expression vector (pCDNA-MALAT1) into Hep-2 cells that had a low endogenous MALAT1 expression level as shown in Fig. 2a. Consequently, comparing with control and pCDNA-NC group, pCDNA-MALAT1 treated Hep-2 cells showed increased MALAT1 mRNA level since 24 h after transfection(*P* < 0.05, Fig. 4e). Western blots analysis indicated that β-catenin, p-β-catenin and NF-κBp65 were increased significantly in pCDNA-MALAT1 Hep-2 cells(*P* < 0.05, Fig. 4f).We next employed the wound-healing assay and transwell assay to further assess the changes of invasion or migration ability by introducing MALAT1 into Hep-2 cell. Wound-healing assay indicated that, pCDNA-MALAT1 transfected Hep-2 cell displayed increased wound healing speed comparing with the control cells(*P* < 0.05, Supplementary Figure 1a,b). The transwell assay with matrigel revealed that in Hep-2 cells after transfection, invaded cell numbers increased significantly(*P*  < 0.05, Supplementary Figure 1c,d). We also used measured western blot to evaluated the changes of EMT markers expression in Hep-2 cell with pCDNA-NC or pCDNA-MALAT1 transfection, The results shown that N-cadherin, Vimentin, Slug, MMP-9 and VEGF expression were all upregulated significantly (*P* < 0.05, Supplementary Figure 1e).These data suggested that MALAT1 might involve in regulating β-catenin and NF-κB signaling pathways in OSCC.

### Inhibition of MALAT1 suppressed tumor growth in Tscca xenograft model

**Figure 5:**
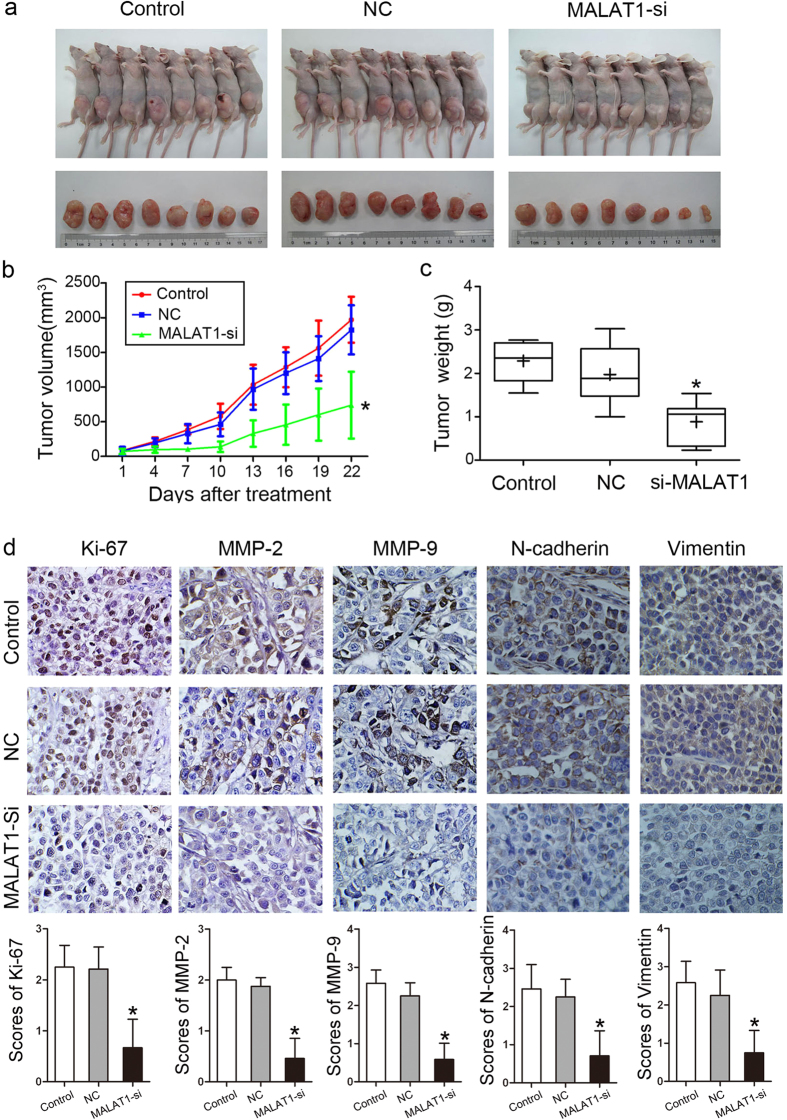
MALAT1 knockdown inhibited tumor growth in a xenograft model *in vivo*. (**a**) The image of Tscca xenograft tumors treated with si-NC and si-MALAT1. (**b**) Tumor growth curve indicated that MALAT1 siRNA injection treatment inhibited significantly Tscca xenograft tumor growth (*P* < 0.05). (c) Average tumor weight of si-MALAT1 group was reduced compared to the control and si-NC groups (*P* < 0.05). (**d**) IHC staining showed the expression of Ki-67, MMP-2, MMP-9, N-cadherin and Vimentin were inhibited in the MALAT1 siRNA-treated Tscca xenograft tumors (original magnification: 200×).

To further verify the role of MALAT1 and to determine the therapeutic potential of targeting MALAT1 in OSCC, we established xenograft tumor models using Tscca cell lines. The tumor weight and tumor growth curve suggested that, MALAT1 inhibition suppressed effectively tumor growth comparing with the control and scramble treated xenograft tumors (*P* < 0.05, Fig. 5a–c). Suppressed Ki67 and MMP2/9 expression by IHC in MALAT1 siRNA treated Tscca tumors suggested the tumor cell proliferation and ECM degradation capability were inhibited significantly (*P* < 0.05, Fig. 5d).And EMT markers including N-cadherin and Vimentin were suppressed in MALAT1 siRNA treated Tscca tumors (*P* < 0.05, Fig. 5d). Because MALAT1 might promote OSCC tumor growth *in vivo*, it may serve as a therapeutic target for treatment.

## Discussion

Recent integrated genomic analysis indicated that abnormal expression of lncRNAs contributes to human carcinogenesis, metastasis, stem cell differentiation, and resistance to chemotherapy or radiation. Generally speaking, lncRNAs, like HOTAIR, function as a scaffold tool to recruit target chromatin-modifying complexes to specific chromatin sites^24^. For example, HOTAIR acts as an onco-lncRNA by recruiting PRC2 complex to catalyze H3K27 triple methylation and thus inhibits transcription of downstream tumor suppressor genes^25^. Targeting HOTAIR could inhibit tumor growth of glioblastoma^26^, breast cancer^27^ and other cancers^28^. Similarly, MALAT1 is overexpressed in many malignancies, including esophageal squamous cell carcinoma^13^, glioblastoma^29^, breast cancer^30^, and prostate cancer^31^. These findings suggest MALAT1 may be an oncogene. In this study, we provided both experimental and clinical evidence to support MALAT1 as a cancer metastasis related molecule and a potential therapeutic target in OSCC.

We validated that MALAT1 was overexpressed in OSCC tumor samples compared with the normal oral mucosa samples, and MALAT1 over-expression confers a shorter survival. Tang H *et al.* reported that no statistical significance of MALAT1 expression was determined in a small OSCC sample^32^. Interestingly, a recent meta-analysis reported that MALAT1 overexpression was correlated with a poor overall survival and the pooled hazard ratio (HR) and corresponding 95% confidence interval (CI) was 1.94 (95% CI 1.59–2.38) in 792 cancer patients^33^. Thus, we believe MALAT1 is a potential prognostic factor in human cancers including OSCC.

Immerging evidence has shown that MALAT1 contributes to cancer invasion and metastasis. In normal cells, MALAT1 interacts with several pre-mRNA splicing factors, such as serine/arginine (SR) splicing factors, and influences alternative splicing of pre-mRNAs by regulating the distribution and activity of SR splicing factors. In colorectal cancer, MALAT1 could disturb the stability of SFPQ/PTBP2 complex, and promote cell proliferation and migration^34^. Shen L reported that MALAT1 could be used as predictor of NSCLC brain metastasis and outcome and MALAT1 promoted brain metastasis of lung cancer by inducing EMT in H1915 cells^35^. MALAT1 can also activate MAPK-ERK signaling to affect cell proliferation and metastasis in gallbladder carcinoma^36^.

Increasing concentration of cisplatin and paclitaxel treatment could suppress the expression of lncRNAs (CDKN2B-AS1, HOTAIR and MALAT1) in laryngeal Hep-2 cells^37^. These findings indicate that MALAT1 is associated with cancer progression by regulating multiple signaling pathways. We proved that MALAT1 knockdown Tca8113 and Tscca cells displayed decreased migration and invasion *in vitro*. Accordingly, the degradation of extracellular matrix was reduced, conferred by down regulation of MMP-2/9 and up regulation of TIMP-3.

NF-κB and β-catenin are believed to be oncogenes that contribute to tumor proliferation and metastasis in many human cancers. For instance, constitutively active p65 subunit of NF-κB inhibited E-cadherin expression by stimulating ZEB-1/2 transcription^38^. Consequent activation of ZEB-1/2 promotes EMT and metastasis. Abnormal activation and nuclear translocation of β-catenin can stimulate gene expression and production of proteins involved in cell transformation by binding to LEF-1/TCF family^39^. Active form of β-catenin stimulates MMPs and VEGF expression to promote cancer invasion and angiogenesis^40^. Our data showed that MALAT1 knockdown cells displayed decreased nuclear accumulation of NF-κBp65, β-catenin and p-β-catenin.

In summary, we demonstrated MALAT1 as an important oncogene like other lncRNAs in OSCC and its expression frequently is upregulated in human tumor samples and cancer cell lines. Functionally, MALAT1 promoted cell migration and invasion via regulating nuclear translocation of NF-κB and β-catenin, and subsequently EMT in OSCC. Further investigation into the molecular mechanism underlying dysregulation of MALAT1 expression in OSCC cells will help to better understand tumor progression of OSCC, identify candidate biomarkers for OSCC prognosis, and guide the development of therapeutic targets for oral cancer.

## Methods

### Tissue samples

A total of 54 OSCC tumor samples from radical resection were randomly collected at Tianjin Medical University Cancer Institute & Hospital during January 2009 to December 2010. Twelve non-tumor normal oral mucosa samples were obtained from Tianjin Medical University Stomatological Hospital. The demographic and clinical data was collected from electric records. All the tissue samples were used for MALAT1 expression examination. All samples were collected with informed consent according to the Internal Review and Ethics Boards of the Tianjin Medical University Medical Ethnic Committee.

### Cell culture and transfection

Human tongue squamous cell carcinoma cell lines Tscca, Tca8113P160, Tca8113 and Hep-2 were purchased from the Institute of Basic Medical Sciences, Chinese Academy of Medical Sciences. Tb3.1 cell line was a gift from the Ninth People’s Hospital Shanghai Jiao Tong University. The cell lines were maintained in MEM or RPMI-1640 (Thermo Scientific, USA) supplemented with 10% fetal bovine serum (FBS, Thermo Scientific, USA)with 5% CO_2_ at 37 °C.

### RNA extraction and qPCR

Total RNA was extracted by using Trizol (Life technology, USA) reagent according to the manufacture’s protocol and reversely transcribed into cDNA using M-MLV Reverse Transcriptase (Life technology, USA). RT-qPCR was performed using Step One Plus system (Applied Biosystems, USA). The primer of MALAT1 was “GACGGAGGTTGAGATGAAGC” (Forward) and “ATTCGGGGCTCTGTAGTCCT” (Reverse) (Life technology, USA). The RT-qPCR procedure was performed under the following conditions: 5 min at 95 °C followed by 40 cycles of 10 sec at 95 °C and 45 sec at 60 °C^41^. All MALAT1 expression data were normalized to GAPDH from the same sample. The Ct value of each target gene was normalized against the Ct value of the reference gene (Ct (MALAT1)—Ct (GAPDH)).

### MALAT1 siRNAs transfection

Double-stranded siRNAs (Sigma-Genosys, USA) were used to knockdown MALAT1 from cells at a final concentration of 20 μmol/L.

### Vector transfection

10 μg MALAT1 full-length expression vector (FulenGen, China) was transfected to 2 × 10^6^ OSCC cells by using Lipofectamine3000 reagent as the manufacturer’s instructions (Invitrogen, USA). The PGL-3 empty vector was used as the control plasmid. 72 hr after transfection, total RNA and cell lysate were collected for qPCR and Western blot analysis.

### Western blot analysis

The MALAT1 knockdown cells were washed twice with ice-cold PBS and treated with lysis buffer (Solarbio, China) or nuclear and cytoplasmic extraction reagent kit (Beyotime, China). Heat-denatured protein samples (25 μg per lane) were resolved by SDS poly-acrylamide gel electrophoresis (PAGE) and transferred to an Immobilon-P membrane (Millipore, Bedford, MA). The membrane was incubated with primary anti-body overnight at 4 °C, 1 h with a secondary antibody at room temperature, followed by ECL reagent (Millipore, Bedford, MA) for chemiluminescent detection. Primary antibody of MMP-2/9, TIMP-3, VEGF, Twist-1, GADPH (Santa Cruz, CA), E/N-cadherin, Zeb-1, Vimentin, Slug, β-catenin/-p and NF-κBp65 (Abcam, UK) were used for WB analysis.

### Wound healing assay

A total of 10,000 Tscca or Tca8113 cells were plated in 6-well plates. When cells grew to confluence at 80%, inserts were then removed with sterile forceps to create a wound by using a 200 μl tip. After removing the cellular debris with PBS, cells were exposed to PBS, Scramble and MALAT1 siRNA for 48 h. Cell migration were perceived by inverted microscope and photographed. The wound area was scaled by Image Pro Plus 5.0 (Olympus, Japan).

### Transwell assay

Cell invasion assay was performed using transwell membranes coated with Matrigel (BD Biosciences). Transfected cells were plated at a density of 3 × 10^4^ cells per well. The lower chamber was filled with 20% FBS. After 48 h, cells remaining in the upper chamber were removed with cotton swabs, while invading cells were fixed with 4% paraformaldehyde (Santa Cruz, USA), stained with crystal violet (Solarbo, China).

### Immunofluorescence staining (IF)

For IF study, MALAT1 knockdown and control cells were incubated with primary antibodies (1:200 dilutions) overnight at 4  °C, followed by incubated with FITC-labeled secondary antibody (1:100 dilutions, Santa Cruz, CA) for 1 h at room temperature. Nuclei were counterstained with DAPI reagent (Life technology, USA). Positive cells were visualized using FV-1000 laser scanning confocal biological microscopes (Olympus, Japan).

### Tscca xenograft tumor model

All animal protocols were approved by Tianjin Medical University Animal Care and Use Committee. The female, SPF grade Nu/Nu nude mice at age of 4 weeks (Vital River Laboratories, China) (n = 10 for each group) were implanted subcutaneously with 5 × 10^6^ Tscca cells as described previously^42^. The tumor volume was measured with a caliper every 3 days using a formula (volume = long diameter × short diameter^2^/2). When the volume of xenograft tumor is approximately 250 mm^3^, the tumor was injected with PBS (25 μL, local injection) or MALAT1 siRNA (30 ng, local injection) every 3 days. After 3 weeks, the mice were sacrificed and the xenograft tumors were removed for formalin fixation and preparation of paraffin-embedded sections.

### Immunohistochemistry (IHC)

For IHC staining, paraffin-embedded tumor slides were deparaffinized, rehydrated and incubated with primary antibodies overnight at 4 °C. The primary antibody against Ki-67, MMP2/9 (Santa Cruz, CA), N-cadherin and Vimentin (Abcam, UK) were used for detection. Then, they were incubated with biotin-labeled secondary antibody (ZhongshanBio Corp., China) for 1h at room temperature and incubated with diaminobenzidine (Zhongshan Bio Corp., China). The results were assessed by measuring both the staining intensity and the number of positive cells. The intensity of the positive reaction was scored, i.e. 0 = negative, 1 = weak, 2 = moderate, and 3 = intense staining. Additionally, staining was scored on a scale of 0–3 according to the percentage of the cells involved: i.e. 0 = 0–5%; 1 = 6–25%; 2 = 26–50%; and 3 = 51–100% positive cells. The scores for the intensity and the percentage of positive cells were multiplied to work out at a weighted score for each case. A score of 0–3 was defined as low expression (−), and scores of 4–9 as high expression (+).

### Statistical Analysis

Data was expressed as means ± S.E. Statistical analysis was performed by ANOVA, χ^2^ test, or Student’s t-test using SPSS18.0 software. Statistical significance was determined as a *P* < 0.05 (*).

## Ethics Statement

Methods used in this study were carried out in accordance with the approved guideline of the Tianjin Medical University Medical Ethnic Committee. All subjects provided written informed consent for participation in this study and a review of their medical records, and provided a sample of tumor tissue sample. We clarified that all the procedures by using the human tissues, including colleting the tissue samples, RNA extraction and qPCR, were carried out in accordance with the Human Tissues Use Guidelines of Tianjin Medical University Medical Ethnic Committee. All experimental animal protocols were carried out in accordance with Experimental Animal regulations of Tianjin Medical University Animal Care and Use Committee.

## Additional Information

**How to cite this article**: Zhou, X. *et al.* Long Non Coding RNA MALAT1 Promotes Tumor Growth and Metastasis by inducing Epithelial-Mesenchymal Transition in Oral Squamous Cell Carcinoma. *Sci. Rep.* **5**, 15972; doi: 10.1038/srep15972 (2015).

## Supplementary Material

Supplementary Information
