# Supplementary material for: Long Non Coding RNA MALAT1 Promotes Tumor Growth and Metastasis by inducing Epithelial-Mesenchymal Transition in Oral Squamous Cell Carcinoma
Source: Sci Rep. 2015 Nov 2;5:15972. doi: 10.1038/srep15972 (PMC4629155; doi:10.1038/srep15972)
Supplement: Supplementary Information [file srep15972-s1.pdf]

Long Non Coding RNA MALAT1 Promotes Tumor Growth and Metastasis by inducing Epithelial-Mesenchymal Transitionin Oral Squamous Cell Carcinoma

Xuan Zhou[1*], Su Liu[1*], Guoshuai Cai[2*], Lingping Kong[1*], TingtingZhang[3], Yu Ren[4], Yansheng Wu[1], Mei Mei[4], LunZhang[1]&Xudong Wang[1]

Supplementary Figure

Overexpression of MALAT1 enhanced OSCC invasion and migration *in vitro*. (a) and (b), pCNDA-MALAT1 transfection significantly increased Hep-2 cell migration determined by wound healing assay (*P*<0.05). (c) and (d), pCNDA-MALAT1 transfection significantly increased Hep-2 cell migration and invasion determined by transwell assays (*P*<0.05). (e), MALAT1 overexpression significantly increased the expression of N-cadherin, Vimentin, Slug, MMP-9 and VEGF in Hep-2 cell determined by western blots analysis (*P*<0.05). GADPH was used as loading control. The gels were crapped, but those gels were run under the same experimental condition.

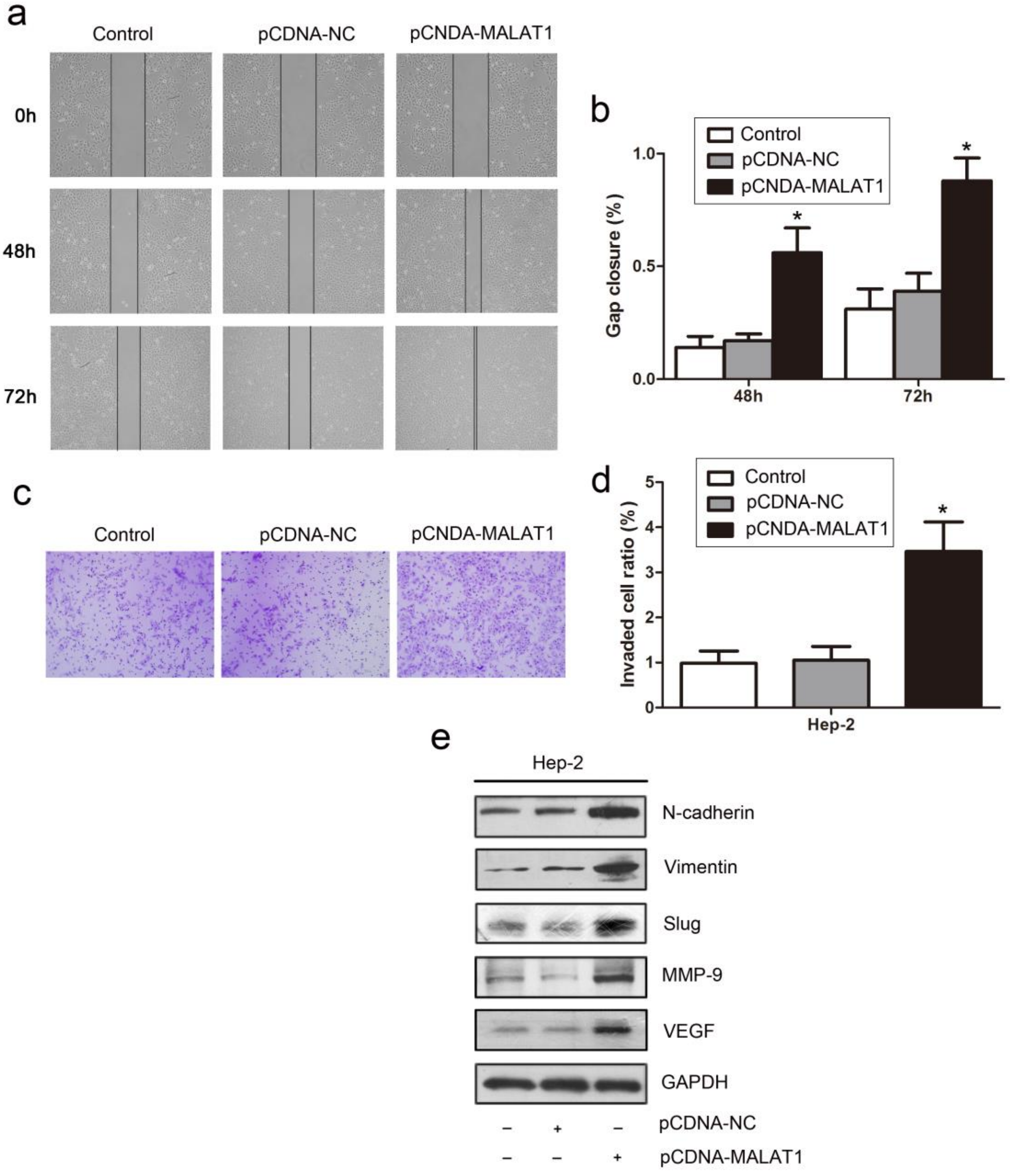
a
Control
pCDNA-NC
pCNDA-MALAT1
0h
48h
72h
b
Control
pCDNA-NC
pCNDA-MALAT1
Gap closure (%)
1.0
0.5
0.0
*
*
48h
72h
c
Control
pCDNA-NC
pCNDA-MALAT1
d
Control
pCDNA-NC
pCNDA-MALAT1
Invaded cell ratio (%)
5
4
3
2
1
0
*
Hep-2
e
Hep-2
N-cadherin
Vimentin
Slug
MMP-9
VEGF
GAPDH
– + – pCDNA-NC
– – + pCDNA-MALAT1
